# No detection of EBV, BKV and JCV in breast cancer tissue samples in Iran

**DOI:** 10.1186/s13104-019-4178-3

**Published:** 2019-03-25

**Authors:** Razieh Dowran, Negar Joharinia, Akbar Safaei, Sahar Bakhtiyarizadeh, Abootaleb Alidadi Soleimani, Rasool Alizadeh, Sara Mir-Shiri, Jamal Sarvari

**Affiliations:** 1 Department of Bacteriology & Virology, School of Medicine, Shiraz University of Medical Sciences, Shiraz, Iran; 2 Department of Pathology, School of Medicine, Shiraz University of Medical Sciences, Shiraz, Iran; 3 Gastroenterohepatology Research Center, Shiraz University of Medical Sciences, Shiraz, Iran

**Keywords:** BKV, JCV, EBV, Breast cancer

## Abstract

**Objective:**

The most common cancer amongst women is breast cancer. Reports on the role of EBV, BKV, and JCV in the development of breast cancer are controversial. Hence, the aim of this study was to determine the frequency of EBV, BKV, and JCV in malignant breast tumors in comparison with benign ones.

**Results:**

A total of 300 breast biopsy tissues were included, of which 150 were malignant and 150 benign. After deparaffinization, tissues were subjected to DNA extraction. β-globin gene was amplified by PCR to evaluate the quality of extracted DNA. In house PCRs assay was performed to detect EBV, JCV, and BKV genome fragment. The mean age of malignant and benign groups was 45.0 ± 9.4 and 35.2 ± 12.1 years old. Out of 150 malignant samples, 146 were ductal, two lobular and two samples both invasive ductal and lobular carcinoma. In the benign group, 96, 52 and two samples were fibroadenoma, fibrocystic, and adenosis types, respectively. Genomic DNA fragment of EBV, BKV, and JCV was not found in any of the malignant and benign breast tissues.

**Conclusion:**

According to our finding, there is the possibility that EBV, BKV, and JCV are not involved in breast cancer pathogenesis.

## Introduction

According to a recent report, there are 17.5 million cancer cases and 8.7 million related deaths worldwide [1]. Among cancers, breast cancer is the most common cancer with around 2.4 million cases in 2015 [1]. In Iran, there are 4000 related deaths and 7000 new cases annually and with the incidence rate is 24 per 100,000 people, as the most common type of cancer amongst Iranian women [2].

Environmental, hereditary and genetic background are considered as the most important factors involved in breast cancer development/progression. Among environmental factors, viral infections appear to be associated with about 15–20% of all cancers [3]. Viruses including Hepatitis B virus (HBV), Epstein Barr virus (EBV), Human polyomavirus (HPV) as well as some polyomaviruses such as Merkel cell polyomavirus (MCP_y_V), BK virus (BKV), and John Cunningham virus (JCV) have been associated with human cancers [4, 5].

EBV role in the pathogenesis of several malignancies, including Burkittlymphoma, nasopharyngeal carcinoma, and gastric cancer has been verified, but its role on breast carcinoma development remains to be determined [3, 6]. EBV resides in B lymphocyte cells with access to breast tissues that leads to infected breast epithelial cells [7]. Hence, there is a possibility that EBV might increase the risk of breast cancer [6]. EBV proteins, expressed in the latent/productive cycle might have an important role in cell transformation. Matrix metallopeptidase 9 induced by BZLF1 forms complexes with P53 and P56, which does not allow infected cells to become apoptosis. Moreover, BRL1 induces E2F, by entering the infected cells to S-phase and proliferation [8]. In addition, late membrane protein1 (LMP1) and 2 mimic cellular receptors continuously active signaling pathways that induce proliferation and survival of the infected cells. Furthermore, miRNAs encoded by EBV have an important role in immune evasion and cell survival [9].

BKV and JCV are members of *Polyomaviruses* family, and human is the sole host of BKV and JCV [10]. Primary infection by JCV and BKV are predominantly asymptomatic and the viruses persist in kidney and B lymphocytes [11]. Since 1953, polyomaviruses have been considered as etiologic agents in cancer, when Gross et al. discovered murine polyomavirus. JCV and BKV contain a double strand DNA (dsDNA) genome, which encodes two oncoproteins called large T antigen (T-Ag) and small t antigen (t-Ag) that are involved in cell transformation [12]. BKV Large T-Ag binds to the tumor suppressor proteins including retinoblastoma-associated protein (RB) and p53 leads to cycle progression as well as apoptosis inhibition [13]. JCV T-Ag activates ataxia-telangiectasia mutated (ATM)/ATM-and Rad3-Related (ATR) mediated G2 checkpoint pathways, causing G2 cell cycle arrest. Moreover, T-Ag can cause DNA damage by inhibiting homologous recombination directed DNA repair through its interaction with IRS1; hence, generating genetic instability in JCV containing cells [14, 15]. In addition, these viruses induce chromosomal aberrations in cells and by a ‘hit and run’ mechanism recruiting the neighboring cells and distant cell proliferation. They also establish latent infection throughout life and their T-Ag is detected in different types of human cancers [4, 10, 16].

Cancer is the third cause of mortality in Iran [17] and between different type of cancer, breast cancer was the second most common cause of death in 2015 [1]. Moreover, the most common types of primary breast cancer in the Iranian population are invasive ductal carcinoma and invasive lobular carcinoma with frequencies of 77.8% and 5.2% [18]. To the best of our knowledge, few studies have investigated the role of EBV, JCV and BKV in breast carcinomas in our region; hence, we conducted this study in the city of Shiraz, Iran.

## Main text

### Subjects and methods

A total of 300 breast tissue biopsies including 150 malignant and 150 benign tissue specimens were collected from Faghihi Hospital and Motahari clinic affiliated to Shiraz University of Medical Sciences. All cases had surgery from January 2008 to December 2015. Tissues were selected according to the pathology reports and had been verified by an expert pathologist. This study was approved by the local Ethics Committee of Shiraz University of Medical Sciences (Approval No. IR.SUMS.REC.1394.S372).

### DNA extraction and qualification

Deparaffinization and DNA extraction were done as previously described [19, 20]. Polymerase chain reaction (PCR) with consensus primers PCO3/PCO4 (Table 1) was performed to amplify β-globin gene fragments to confirm the quality of extracted DNA. PCR for amplifying β-globin gene was performed as previously described [20]. A total of 321 samples were selected for assessment, of which 21 samples were excluded based on the ß-globin gene negative PCR result.

**Table 1:** The Sequences and other characteristics of primers used in this study

Target	Primers	5′ to 3′ sequence	Product size, bp
β-Globin	PCO3	5′-ACACAACTGTGTTCACTAGC-3′	110
	PCO4	5′-CAACTTCATCCACGTTCACC-3′	
JCV	Large T F	5′-TGAGGAATGCATGCAGATCTAC-3′	248
	Large T R	5′-TTTGCAGGGCATTTTGTTTTTTAC-3′	
BKV	BRP-1	5′-TTGAGAGAAAGGGTGGAGGC-3′	265
	BRP-2	5′-GCCAAGATTCCTAGGCTCGC-3′	
EBV	EBV F	5′-TACTCCTTACTATGTTGTG-3′	298
	EBV R	5′-CCTTGCCTAATATCCTAC-3′	

### EBV genome detection

To investigate the presence of the EBV genome fragment, AlleleID 7 software was used. The designing primers, specific for the BHRF1 region of the virus are shown in Table 1. EBV genome amplification was done on samples that were positive for the β-globin gene. PCR mix reaction components were 2×PCR master mix red (Ampliqon, Denmark) and 0.4 µM of each primer in 25 µl final volume. PCR tests for BHRF1 coding region of EBV was performed as follow: 10 min initial denaturation at 95 °C, 45 cycles of denaturation at 95 °C for 45 s, annealing at 57.6 °C for 45 s, extension at 72 °C for 45 s and final extension at 72 °C for 10 min. PCR products were then loaded into 2% agarose gel and visualized under UV light. The DNA extracted from the B-95 cell line (Institute pasture, Tehran, Iran) was used as a positive control during each run.

### JCV genome detection

Also, positive breast tissue samples for the β-globin gene underwent PCR to detect JCV. Specific primers targeting JCV Large T antigen were selected from previous studies (Table 1) [21]. PCR was performed in a mix containing 1 mM MgCl2, 200 μM dNTPs, 1U Taq DNA polymerase (CinnaGene, Tehran, Iran) and 1 μM each specific primer in a total volume of 25 μL (Table 1). PCR program was as follow: 10 min initial denaturation at 95 °C, 50 cycles of denaturation at 95 °C for 1 min, annealing at 48 °C for 1 min, extension at 72 °C for 2 min, and final extension at 72 °C for 8 min. Then PCR products were loaded into 2% agarose gel and visualized under UV light. A positive control for JCV was provided by Keyvan Virology Laboratory (Tehran, Iran).

### BKV genome detection

We also performed PCR to detect BKV in breast tissue samples as previously describe [21].

## Results

### Demographic and pathological result

Out of 150 malignant samples, 146 (97.3%) ductal, two (1.3%) lobular and the other two (1.3%) were both invasive ductal and lobular carcinoma. The mean age of participants in malignant and benign groups was 45.0 ± 9.4 SD and 35.2 ± 12.1 SD. Out of 150 malignant samples 35 (23.3%), 67(44.7%) and 48 (32%) were at stage I, II and III, respectively based on the American Joint Committee on Cancer system. In the case of benign samples, 96 (64%) were fibroadenoma, 52 (34.7%) were fibrocystic and 2 (1.3%) were adenosis type.

### PCR for detection of EBV, JCV, and BKV

**Fig. 1:**
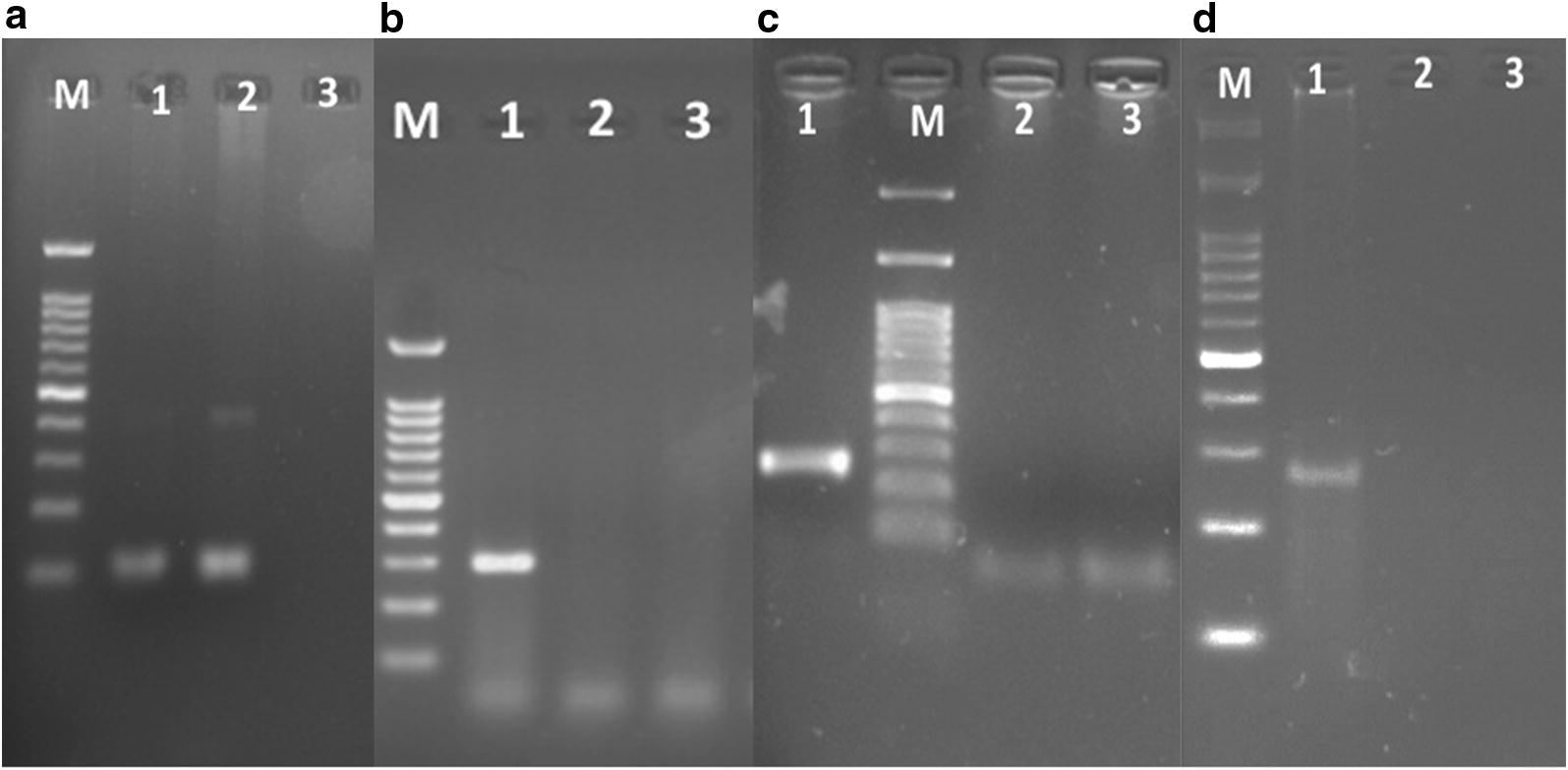
Photographs of gel electrophoresis. M: 100 bp DNA size marker; lanes 1: positive control; lanes 2: sample; lanes 3: Negative control. **a** PCR analysis of DNA samples extracted from breast tissue using β- globin primers. **b** PCR analysis of DNA samples extracted from breast tissue using EBV common primers. **c** PCR analysis of DNA samples extracted from breast tissue using type-specific primers for JCV. **d** PCR analysis of DNA samples extracted from colorectal tissue using type-specific primers for BKV

In our study, none of the cancerous and benign breast tumor tissues were positive for the presence of EBV, JCV and BKV DNA fragment as shown in Fig. 1.

## Discussion

Breast cancer is the main type of cancer amongst women [1]. Certain risk factors including age, **e**arly menstrual period, late or no pregnancy, menopause after age 55, not being physically active, taking oral contraceptives, family history of breast cancer, alcohol consumption, and smoking, have been proposed as risk factors for breast cancer development/progression [22].

The result of our study showed that none of the cancerous and benign samples were positive for EBV. In line with this finding, Perrigoue et al. found that all of the 45 tumor biopsies samples were negative for EBV DNA fragment [23]. Also, in Iran, Kadivar et al. showed that all cancerous and noncancerous tissue samples were negative for EBV DNA and protein [24]. On the other hand, Louise et al. reported an association between EBV and breast cancer [25]. Moreover, Bonnet et al. showed a higher frequency of EBV in cancerous tissue in comparison with healthy controls [26]. In addition, Mazouni et al. revealed that the frequency of EBV in breast cancer tissue was higher in more aggressive phenotype tumors [27]. Also, Zekri et al. showed the association of EBV with breast cancer [28]. Furthermore, in a study by Antonsson et al. 10% (5/54) of breast tumor samples were positive for EBV genome [29]. In Iran, in a case–control study, EBV was detected in breast cancer tissues, while all the control samples were negative for EBV [30].

Finally, the two meta-analysis by Huo et al. and Amarante et al. showed a significant association between EBV and breast cancer [31, 32]. A reason to investigate the association between EBV and breast cancer is that EBV infection confers resistance to an anticancer chemotherapy drug [8].

Although some reports showed the presence of EBV in breast cancer, others reported the absence of EBV genome/protein in breast cancer tissue. This difference might be due to limitation of the detection methods. As evidence, low levels of EBV genome was found in breast tumors by TaqMan real-time PCR while negative result was obtained in studies with less sensitive methods [33]. Also ‘hit and run’ mechanism was proposed as EBV oncogenesis; hence, the viral genome might be lost during malignant cell division [7].

In the case of JCV, none of the 150 malignant and 150 benign samples were positive for the JCV genome. In agreement with our results, Antonsson et al. reported that none of the 54 breast tumor samples were positive for JCV [29]. On the other hand, Hachana et al. reported that 23% (28/123) of breast cancer tissues were infected with JCV [34]. In addition, Moore et al. tested the milk from women with and without breast cancer history and found higher virus particles in the milk of women with familial history of breast cancer [35].

In our study, none of the malignant and benign breast tissues were positive for BKV genome fragment. In line with our result, Hachana et al. reported that BKV was not detected in breast carcinoma tissues of Tunisian patients [34]. Antonsson et al. also showed that none of the 54 breast tumor samples were positive for BKV [29]. Generally, low JCV and BKV DNA detection in tumors can be explained as the transient effect of JCV in cellular transformation, which is called “hit and run’’ transformation mechanism [36]. Different laboratory methods and/or sample size, population, geographic, and differences in the detection limit of the methods inevitably might explain the controversy. In conclusion, the results of our study revealed that the DNA fragment of EBV, JCV, and BKV was not detected in any of the malignant and benign breast samples. Therefore, it can be said that there is no association between EBV, JCV, and BKV infection and breast cancer. To identify the role of these viruses in breast cancer, more investigations with larger sample sizes and more sensitive methods are recommended.

## Limitations

It can be noted that the limitations of our study were working on formalin-fixed paraffin-embedded tissue specimens as well as using conventional PCR assay, which can be resolved in similar future studies, by performing real-time PCR on fresh biopsy specimens.

## Abbreviations

HBV: Hepatitis B virus
HCV: Hepatitis C virus
EBV: Epstein Barr virus
HPV: human papillomavirus
MCP_y_V: Merkel cell polyomavirus
BKV: Thea Brennan-Krohn virus
JCV: John Cunningham virus
dsDNA: double strand DNA
LMP1: late membrane protein1
AIDS: acquired immune deficiency syndrome
RB: retinoblastoma-associated protein
ATM: ataxia-telangiectasia mutated
PCR: polymerase chain reaction
